# Unraveling the Structure of Meclizine Dihydrochloride with MicroED

**DOI:** 10.1002/advs.202306435

**Published:** 2023-12-03

**Authors:** Jieye Lin, Johan Unge, Tamir Gonen

**Affiliations:** ^1^ Department of Biological Chemistry University of California 615 Charles E. Young Drive South Los Angeles CA 90095 USA; ^2^ Department of Physiology University of California 615 Charles E. Young Drive South Los Angeles CA 90095 USA; ^3^ Howard Hughes Medical Institute University of California Los Angeles CA 90095 USA

**Keywords:** Meclizine (Antivert, Bonine), Microcrystal electron diffraction (MicroED), Molecular docking, Protein‐drug interactions, Racemic crystal

## Abstract

Meclizine (Antivert, Bonine) is a first‐generation H1 antihistamine used in the treatment of motion sickness and vertigo. Despite its wide medical use for over 70 years, its crystal structure and the details of protein‐drug interactions remained unknown. Single‐crystal X‐ray diffraction (SC‐XRD) is previously unsuccessful for meclizine. Today, microcrystal electron diffraction (MicroED) enables the analysis of nano‐ or micro‐sized crystals that are merely a billionth the size needed for SC‐XRD directly from seemingly amorphous powder. In this study, MicroED to determine the 3D crystal structure of meclizine dihydrochloride is used. Two racemic enantiomers (R/S) are found in the unit cell, which is packed as repetitive double layers in the crystal lattice. The packing is made of multiple strong N‐H‐Cl^−^ hydrogen bonding interactions and weak interactions like C‐H‐Cl^−^ and pi‐stacking. Molecular docking reveals the binding mechanism of meclizine to the histamine H1 receptor. A comparison of the docking complexes between histamine H1 receptor and meclizine or levocetirizine (a second‐generation antihistamine) shows the conserved binding sites. This research illustrates the combined use of MicroED and molecular docking in unraveling elusive drug structures and protein‐drug interactions for precision drug design and optimization.

## Graphical abstract

Meclizine (Antivert, Bonine) is an H1 antihistamine used in the treatment of motion sickness and vertigo for over 70 years. The 3D structure of meclizine dihydrochloride is first determined by microcrystal electron diffraction (MicroED) after direct analysis of its seemingly amorphous powder. Molecular docking reveals the binding mechanism of meclizine to the histamine H1 receptor.

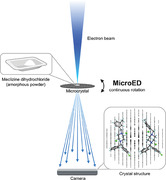

Meclizine, marketed as “Antivert” or “Bonine”, is a first‐generation H1 antihistamine used in the treatment of motion sickness and vertigo.^[^ ^1^, ^2^, ^3^ ^]^ Meclizine is chemically similar to other piperazine‐class H1 antihistamines, such as cyclizine, buclizine, cetirizine, hydroxyzine, levocetirizine, and quetiapine.^[^ ^4^ ^]^ It consists of phenyl, chlorophenyl, and piperazine groups connected by a chiral carbon, with the methylbenzyl group linked on the other side of the piperazine ring (**Figure** **1**A). The crystal structures of the piperazine‐class antihistamines were mostly solved by single‐crystal X‐ray diffraction (SC‐XRD) over the last several decades: cyclizine (1980),^[^ ^5^ ^]^ quetiapine (2005),^[^ ^6^ ^]^ cetirizine (2015),^[^ ^7^ ^]^ and buclizine (2020).^[^ ^8^ ^]^ The disordered hydroxyzine model was derived by powder X‐ray diffraction (PXRD) in 2019.^[^ ^9^ ^]^ The conventional SC‐XRD encounters difficulties in obtaining large crystals from powdery substances,^[^ ^10^ ^]^ and solving PXRD structure can be challenging due to peak overlapping and broadening.^[^ ^11^ ^]^ Therefore certain challenging crystal structures of piperazine‐class H1 antihistamines were left unattainable for decades. The structure of meclizine remained elusive for more than 70 years despite its widespread medical use, ranking 142 as the most prescribed medicine in 2020 with more than 4 million prescriptions.^[^ ^12^ ^]^ Nuclear magnetic resonance (NMR) has recently been shown to be able to characterize the atomic structure of nano‐crystalline to amorphous materials, which are not suitable for single crystal diffraction methods. This was possible in combination with a large number of molecular dynamics simulations and extensive calculations to corroborate and interpret the experimental results.^[^ ^13^, ^14^ ^]^

**Figure 1:**
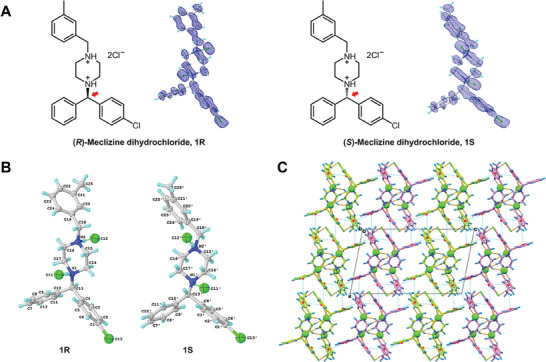
A) Chemical structure and 2F_o_‐F_c_ map (0.5 e Å^−3^) of meclizine dihydrochloride (**1R**/**1S**). B) Chemical notations of **1R** and **1S**. **1R** was labeled with atom type and numbers, and **1S** was labeled with atom type and primed numbers. C) Crystal packing diagram of **1R/1S**, viewed along the *b* axis. **1R** was highlighted in green, **1S** was highlighted in violet. Hydrogen bonding interactions were represented by the dashed lines in orange, and pi‐stacking interactions were represented by the dashed line in cyan. Cl^−^ anions were highlighted in spacefill style. See details in Figure 2; Tables S3, and S4, Supporting Information.

Microcrystal electron diffraction (MicroED) has emerged as a revolutionary technique that overcame the crystal size limitations of SC‐XRD.^[^ ^15^, ^16^ ^]^ It enables the analysis of nanocrystals directly from seemingly amorphous powder, which is merely a billionth the size needed for SC‐XRD. The advent of MicroED has provided an alternative route for the structure elucidation of antihistamines with previously unknown crystal structures. For example, MicroED recently succeeded in solving the structure of levocetirizine, a compound whose crystal structure was unknown for 16 years after its initial medical use.^[^ ^17^ ^]^

The histamine H1 receptor is a member of the rhodopsin‐like G protein‐coupled receptor family that presents in various tissues, including smooth muscle, endothelial cells, and neurons in the central nervous system (CNS).^[^ ^18^, ^19^, ^20^ ^]^ Activation of this receptor by its biological agonist (histamine) regulates allergic responses, while the H1 antihistamine drugs reduce the receptor's activity by binding and blocking the histamine interaction as an inverse agonist.^[^ ^18^, ^19^, ^20^ ^]^ First‐generation antihistamines like meclizine involve several nonselective interactions with other receptors in the CNS, causing various adverse effects, such as drowsiness, dry mouth, and fatigue.^[^ ^21^ ^]^ While second‐generation antihistamines like levocetirizine minimized adverse effects by reducing brain penetration and increasing binding selectivity.^[^ ^22^ ^]^ In this study, we used MicroED to determine the atomic structure of meclizine dihydrochloride. Molecular docking was then employed to analyze the binding between meclizine and the histamine H1 receptor, revealing its antihistamine mechanism and conformational changes between the drug formulation state and its biologically active state.

The meclizine dihydrochloride sample preparation for MicroED followed the previously described procedure (see details in the Supporting Information).^[^ ^23^ ^]^ The grid containing the crystals was examined using the 200 kV Thermo Fisher Talos Arctica Cryo‐TEM with ≈0.0251 Å wavelength. The microscope was equipped with a CetaD CMOS camera and EPUD software.^[^ ^24^ ^]^ The crystal thickness played a crucial role in obtaining optimal diffraction, so crystals were initially screened using imaging mode (LM 210×) for a grid atlas. Only crystals with a certain brightness contrast were thin enough and were selected for further analysis (Figure S1, Supporting Information). The eucentric height for each crystal was manually calibrated at low magnification (SA 3400×) to ensure proper centering during the continuous rotation. For data collection, a 70 µm C2 aperture and a 50 µm selected area (SA) aperture were utilized to reduce background noise and achieve a near‐parallel 1.4 µm beam size. The crystal was found to be very sensitive to radiation damage, for example crystal lattice was damaged after 40 s even using the weakest spot size 11 under microprobe mode (0.0098 e^−1^ Å^−2^ s^−1^), therefore an increased rotation rate of ≈2 s over a smaller angular range of 80° (−40° to +40°), with an exposure time of 0.5 s per frame were used in order to minimize the total electron doses to 0.39 e^−1^ Å^−1^.^[^ ^2^ ^]^ The MicroED movies were converted from mrc format to smv format using the mrc2smv software (available freely at https://cryoem.ucla.edu/microed).^[^ ^24^ ^]^ High‐quality datasets were indexed and integrated using XDS,^[^ ^25^, ^26^ ^]^ resulting in a completeness of over 55% for each dataset (Table S1, Supporting Information). The completeness was increased to 80.7% after scaling and merging data from two individual crystals (Table S2, Supporting Information). The intensities were converted to SHELX hkl format using XDSCONV.^[^ ^26^ ^]^ The MicroED structure was solved ab initio using SHELXT^[^ ^27^ ^]^ at a resolution of 0.96 Å. The MicroED structure of meclizine dihydrochloride contains two enantiomers, designated as **1R**/**1S**, and was determined to be a centrosymmetric monoclinic space group P2_1_/c, with the unit cell of a = 14.39 Å, b = 7.19 Å, c = 24.52 Å, α = 90.000°, β = 101.958°, γ = 90.000°.^[^ ^28^ ^]^ Subsequent refinement using SHELXL^[^ ^29^ ^]^ yielded a final R_1_ value of 17.89% (for more refinement statistics please see Table S2, Supporting Information). The positions of heavier atoms were accurately determined from the charge density map (Figure 1A). Since not all hydrogen (H) atoms could be located at this resolution, their positions were refined using a combination of constrained and free approaches.

The two enantiomers, **1R and 1S** (See notations in Figure 1B), are packed as repetitive double layers (**1R**‐**1R** or **1S**‐**1S**) in the crystal lattice (Figure 1C). In the *b*‐axis, those layers are strengthened by various internal hydrogen bonding. Using the **1R**‐**1R** layers for example, hydrogen bond interactions can be categorized into three groups (**Figure** **2**A and Table S3, Supporting Information): 1) Two charge‐assisted hydrogen bonds N1‐H‐Cl1 and N2‐H‐Cl2 are along the *b* axis, with distances at ≈3.0 Å. 2) Seven C‐H‐Cl1 and three C‒H‐Cl2 hydrogen bonds that formed around Cl^−^ anions are along the *a* and *b* axes. These weak C‐H‐Cl^−^ hydrogen bonds, ≈3.5–3.7 Å, were found between Cl^−^ anions and atoms in aromatic phenyl rings (C5, C9, C3, C24), piperazine rings (C14, C15), and alkane chains (C13, C18) of four surrounding **1R** molecules.^[^ ^30^ ^]^ (3) A weak C25‒H‐Cl3 hydrogen bond is established between methylbenzyl ring (C25) and chlorophenyl ring (Cl3).^[^ ^30^ ^]^ The same hydrogen bond geometry was found in **1S**‐**1S** layers, albeit with different symmetries (Figure 2B; Table S3, Supporting Information). The packing along *a* and *c* axes is maintained by numerous pi‐stacking interactions within the lattice, where three aromatic phenyl rings in a single **1R** or **1S** molecule can interact with up to fourteen aromatic rings from the surrounding ten molecules. The chlorophenyl ring 1 in **1R** for example interacts with rings 4–7 in parallel‐displaced mode, the phenyl ring two interacts with rings 8–10 in T‐shaped mode, and the methylbenzyl ring three interacts with rings 11–17 in a combination of parallel‐displaced or T‐shaped mode (Figure 2C; Table S4, Supporting Information).^[^ ^31^ ^]^ The identical pi‐stacking geometry was observed in **1S** but interacted with different molecules (Figure 2D; Table S4, Supporting Information). Such interactions reinforce the packing within the **1R**‐**1R** or **1S**‐**1S** layers and establish connections between **1R** and **1S** molecules, further extending the crystal packing along the *a* and *c* axes. However, most of these interactions are remarkably weak, resulting in a fragile crystal lattice susceptible to external forces.^[^ ^30^, ^31^ ^]^

**Figure 2:**
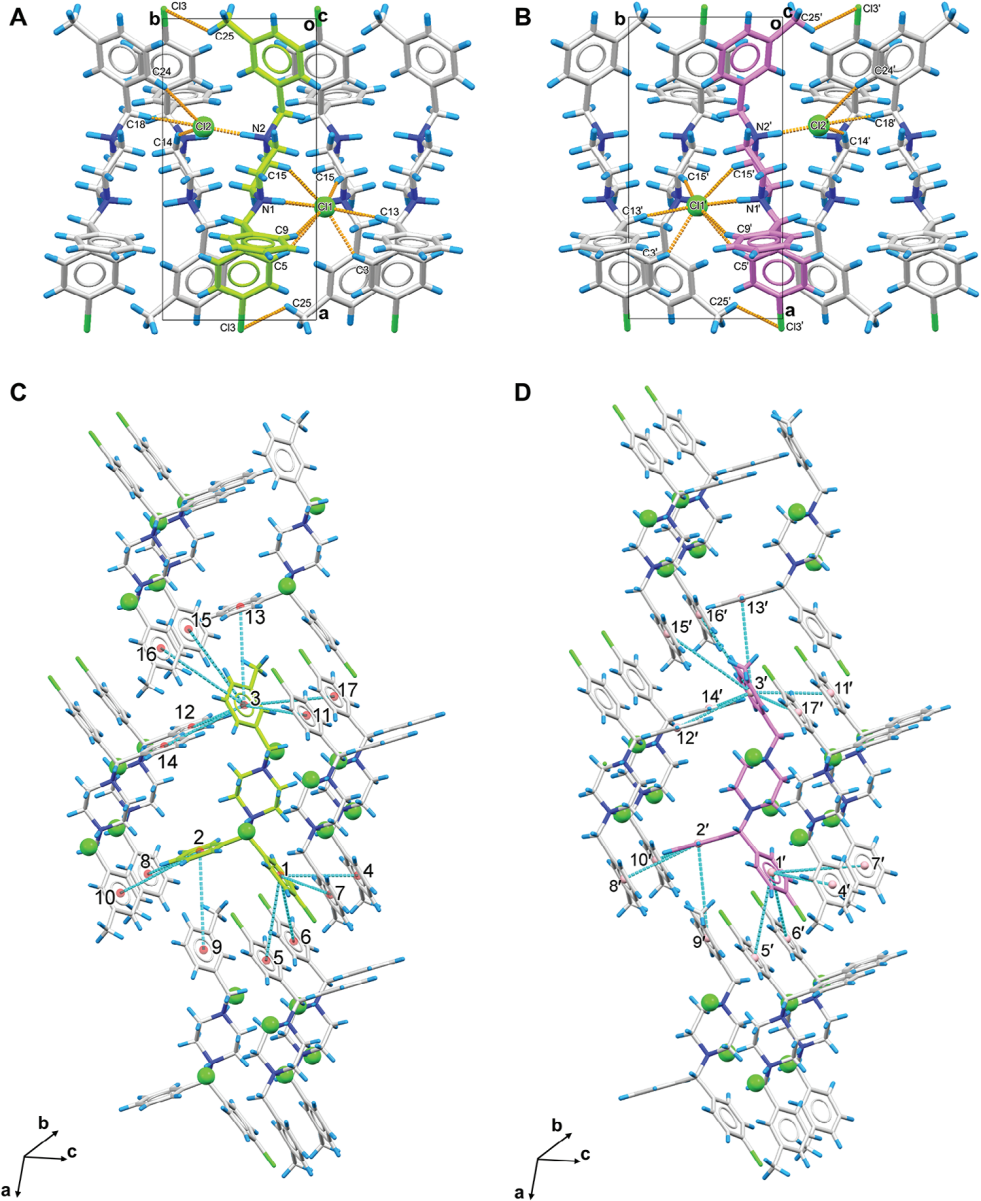
Hydrogen bonding and pi‐stacking interactions in meclizine dihydrochloride (**1R/1S**) crystal packing. A–B) Hydrogen bonding interactions in **1R** and **1S**, respectively, viewed along the *c* axis; C–D) Pi‐stacking interactions in **1R** and **1S**, respectively, showing strong and moderate interactions with ten molecules in the surroundings. **1R** was colored in green, **1S** was colored in violet. Hydrogen bonding interactions were represented by the dashed lines in orange, and pi‐stacking interactions were represented by the dashed line in cyan. Cl^−^ anions were highlighted in spacefill style.

Like the related piperazine‐class antihistamines, meclizine dihydrochloride (**1R**/**1S**) contains phenyl, chlorophenyl, and piperazine rings, which are connected by a chiral carbon, and the methylbenzyl ring is linked to the other side of piperazine ring (Figure 1). The crystal structure of **1R**/**1S** depicts the structure in its drug formulation state, serving as the initial reference prior to its transition into a biologically active conformation. Inspection of **1R**/**1S** showed similar C‐C bond lengths without any substantial stretch or compression, varying from 1.47 to 1.57 Å (except phenyl rings), and the C‐N bond lengths range from 1.40 to 1.55 Å. The C‐C‐N or C‐N‐C bond angles maintain a nearly perfect sp^[^ ^3^ ^]^ geometry, with an average value of 112.0 ± 4.0° in **1R**/**1S**. As for the heterocyclic piperazine rings, the N1‒C14‒C15‒N2/N1′‒C14′‒C15′‒N2′ and N1‒C16‒C17‒N2/N1′‒C16′‒C17′‒N2′ torsion angles are ± 57.8° and ± 63.7° in **1R**/**1S**, respectively. The distances between N1/N1′ and N2/N2′ atoms to the mean plane of C14‒C15‒C16‒C17/C14′‒C15′‒C16′‒C17′ are 0.72 Å and 0.65 Å, respectively. The piperazine rings maintain an almost perfect chair conformation due to the near 60° N‐C‐C‐N torsion angles and comparable distances between the C‐C‐C‐C planes and nitrogen atoms. The same conformation was also observed in other piperazine‐class antihistamine structures such as buclizine monohydrochloride monohydrate Cambridge Structural Database (CSD) entry: HUQVAT,^[^ ^8^ ^]^ and levocetirizine dihydrochloride (CSD entry: KIMDOD),^[^ ^17^ ^]^ suggesting the rigid conformation of piperazine ring, which is unaffected by different charge states.

In **1R**/**1S**, five bonds corresponding to torsion angles α (N1‒C13‒C10‒C9/ N1′‒C13′‒C10′‒C9′), β (N1‒C13‒C4‒C5/N1′‒C13′‒C4′‒C5′), θ (C10‒C13‒N1‒C17/C10′‒C13′‒N1′‒C17′), ω (N1‒C13‒C10‒C9/N1′‒C13′‒C10′‒C9′) and γ (C13‒C10‒C9‒C20/C13′‒C10′‒C9′‒C20′) have a relatively high degree of rotational freedom and can significantly influence the overall molecular conformation (Figure S2, Supporting Information). The orientation of phenyl and chlorophenyl rings is determined by the *α* and *β* torsion angles, which are ≈± 47° (staggered‐like), the piperazine ring was controlled by both *θ* and *ω* torsion angles, with values ≈± 62° and ± 64° (staggered). The γ torsion angle is ± 79°, which manifested a staggered‐like conformation and positioned the methylbenzyl ring in the direction of the phenyl group (Figure S2, Supporting Information). As previously mentioned, these torsions may be critical structural parameters in the drug formulation state but may not be important in its biologically active state upon interaction with the receptor. The latter may require a substantial conformational change.

The H1 antihistamine **1R**/**1S** acts as an inverse agonist, which inhibits the interaction between the agonist (histamine) and the histamine H1 receptor.^[^ ^18^, ^19^, ^20^ ^]^ However, the structural details of their binding mechanism remained unclear. Molecular docking of histamine H1 receptor in complex with **1R** or **1S** helped uncover the binding mechanism and the conformation changes between the drug formulation state and receptor‐bound biologically active state of meclizine. A comparative study of the docking complexes between the receptor and **1R**, **1S**, or levocetirizine (a second‐generation antihistamine) identified conserved binding sites. In the setup for molecular docking, the enantiomerically pure ligand structure of **1R**, **1S**, or levocetirizine (CSD entry: KIMDOD)^[^ ^17^ ^]^ was directly obtained from their MicroED structures, with the polar H atoms removed (see details in the Supporting Information). All active torsion angles, including α, β, θ, ω, and γ, were rendered rotatable during the docking. The cryo‐EM structure of the histamine H1 receptor (Protein Data Bank (PDB) entry: 7DFL) was used as a rigid model.^[^ ^32^ ^]^ The molecular docking was conducted by AutoDock Vina 1.1.2^[^ ^33^, ^34^ ^]^ using an 18.75 Å × 18.75 Å × 18.75 Å grid box with 0.375 Å spacing, centered at the experimentally determined ligand (histamine) position (see Figure S3, Supporting Information).

The cryo‐EM structure of the complex formed between the histamine H1 receptor and its agonist (histamine) revealed interactions within four specific transmembrane helices (TMs): **I**, **II**, **III** and **V**. These interactions involved: 1) Two weak hydrogen bonds between Asp107 and Tyr458 residues and histamine's ethylamine group; 2) Three strong hydrogen bonds between Thr112, Asn198, Tyr431, and histamine's imidazole ring (see **Figure** **3**A; Table S5; Supporting Information).

**Figure 3:**
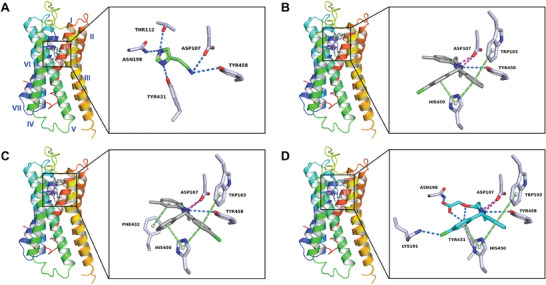
Protein–drug interaction diagram of a complex between histamine H1 receptor and A) histamine, B) **1R**, C) **1S,** and D) levocetirizine. Histamine was colored green, **1R,** and **1S** were colored in grey, and levocetirizine was colored in light blue. Hydrogen bonding and halogen bonding interactions were colored by the dashed line in marine, pi‐stacking, and pi‐cation interactions were colored by the dashed line in lime, and salt bridges were colored by the dashed line in magenta. Seven transmembrane α‐helical segments (**I**–**VII**) in histamine H1 receptor were colored from red to blue, see the overall view in Figure S3 (Supporting Information).

The molecular docking analysis of the histamine H1 receptor complexed with **1R** or **1S** involved some of the same histamine binding residues but also suggested additional binding residues at adjacent regions. The major interactions between the receptor and **1R** involve residues from transmembrane helices (TMs) **I**, **V** and **VI**, including 1) One or two salt bridges between Asp107 and the protonated piperazine ring in **1R**; 2) Weak hydrogen bond between Tyr458 and the piperazine ring in **1R**; 3) Pi‐stacking interactions involving His450, Trp103, and the phenyl or chlorophenyl ring in **1R**; 4) Up to seven hydrophobic interactions involving Tyr, Ile, Trp, and Phe residues in TMs **III** and **V**, stabilizing the orientation of the methylbenzyl ring toward the left. These conformation changes are mainly driven by the rotation of the *θ* torsion angle by more than 100°, resulting in the piperazine ring being nearly perpendicular to the plane formed by phenyl and chlorophenyl rings (Figure S2, Supporting Information). The enantiomer **1S** displayed interactions similar to **1R**, with an additional pi‐stacking interaction between Phe432 and the methylbenzyl ring, possibly enhancing its binding affinity. These conformational shifts were led primarily by rotations of the θ, ω, γ torsions in **1S**, reorienting the phenyl and chlorophenyl rings, yet maintaining the piperazine and methylbenzyl rings in a position analogous to **1R** (Figure S2, Supporting Information).

Levocetirizine, a second‐generation antihistamine, exhibits enhanced binding selectivity and minimal brain penetration compared to **1R**/**1S**.^[^ ^35^ ^]^ The molecular docking analysis of the histamine H1 receptor complexed with levocetirizine showed the conserved binding sites of TRP103, Asp107, His450, and Tyr458, which were consistent with those for **1R**/**1S** (Figure 3D). However, the binding interactions with levocetirizine were stronger and more closely resembled histamine than **1R**/**1S**. For example, the strong hydrogen bonds between Asn198, Tyr431, and the levocetirizine's ethoxyacetic acid group; The robust halogen bond between Lys191 and the levocetirizine's chlorophenyl group, which has been validated in the biochemical binding analysis in the literature.^[^ ^36^ ^]^

In conclusion, we determined the elusive 3D structure of meclizine dihydrochloride (**1R**/**1S**) for the first time using the innovative MicroED technique. This achievement is particularly noteworthy due to meclizine's extensive medical use for over 70 years. Our study not only detailed the crystal packing but also directly determined the 3D structure from its drug formulation state. Using molecular docking, we probed the binding mechanism of meclizine to the histamine H1 receptor, identifying essential contacts within the active sites and revealing the drug's conformational changes between its drug formulation state and its biologically active state. This research serves as a foundation for understanding the binding mechanism of H1 antihistamines to their receptors, employing a combined approach of MicroED and molecular docking. This approach could be used for future precision drug design and optimization pipelines. The study highlights the expanded possibilities in structure determination afforded by advanced techniques like MicroED with its unmatched efficiency in unraveling the structures of nano‐ or micro‐scale drug crystals directly from what appears to be amorphous powder. When combined with other characterization methods like SC‐XRD,^[^ ^10^ ^]^ PXRD,^[^ ^11^ ^]^ and NMR^[^ ^13^, ^14^ ^]^ and molecular docking the tool kit for structure determination and characterization grows, and more avenues for efficient drug optimization become available, facilitating breakthroughs for samples that remained unattainable for decades.

## Conflict Of Interest

The authors declare no conflict of interest.

## Supporting information

Supporting Information

## Data Availability

The data that support the findings of this study are available in the supplementary material of this article.

## References

[1] T. Wibble , J. Engström , L. Verrecchia , T. Pansell , Br. J. Clin. Pharmacol. 2020, 86, 1510. 32077140 10.1111/bcp.14257 PMC7373708

[2] B. Cohen , Arch. Neurol. 1972, 27, 129. 4339240 10.1001/archneur.1972.00490140033006

[3] R. D. Shih , B. Walsh , B. Eskin , J. Allegra , F. W. Fiesseler , D. Salo , M. Silverman , J. Emerg. Med. 2017, 52, 23. 27789115 10.1016/j.jemermed.2016.09.016

[4] R. Vardanyan , V. Hruby , *in* Synthesis of Best‐Seller Drugs, (Eds.: R. Vardanyan , V. Hruby *)*, Academic Press, Boston, 2016, pp. 247–263.

[5] V. Bertolasi , P. A. Borea , G. Gilli , M. Sacerdoti , Acta Crystallogr. B. 1980, 36, 1975.

[6] K. Ravikumar , B. Sridhar , Acta Crystallogr. E. 2005, 61, o3245. 10.1107/S0108270104028926 15640592

[7] J. Majumder , J. Deb , A. Husain , S. S. Jana , P. Dastidar , J. Mater. Chem. B 2015, 3, 6634. 32262799 10.1039/c5tb00676g

[8] M. Bitencourt , O. M. M. S. Viana , A. L. M. Viana , J. T. J. Freitas , C. C. De Melo , A. C. Doriguetto , Int. J. Pharm. 2020, 589, 119840. 32890657 10.1016/j.ijpharm.2020.119840

[9] J. A. Krueger , J. A. Kaduk , A. M. Gindhart , T. N. Blanton , Powder Diffr. 2019, 34, 66.

[10] K. Diederichs , M. Wang , Protein Crystallogr. Methods Protoc. 2017, 1607, 239. 10.1007/978-1-4939-7000-1_10 28573576

[11] K. D. M. Harris , M. Tremayne , B. M. Kariuki , Angew. Chem., Int. Ed. 2001, 40, 1626. 10.1002/1521-3773(20010504)40:9<1626::aid-anie16260>3.0.co;2-7 11353468

[12] S. Kane , ClinCalc DrugStats Database 2020, 20.

[13] M. Cordova , P. Moutzouri , S. O. Nilsson Lill , A. Cousen , M. Kearns , S. T. Norberg , A. Svensk Ankarberg , J. Mccabe , A. C. Pinon , S. Schantz , L. Emsley , Nat. Commun. 2023, 14, 5138. 37612269 10.1038/s41467-023-40853-2 PMC10447443

[14] M. Balodis , M. Cordova , A. Hofstetter , G. M. Day , L. Emsley , J. Am. Chem. Soc. 2022, 144, 7215. 35416661 10.1021/jacs.1c13733 PMC9052749

[15] D. Shi , B. L. Nannenga , M. G. Iadanza , T. Gonen , eLife 2013, 2, e01345. 24252878 10.7554/eLife.01345 PMC3831942

[16] B. L. Nannenga , T. Gonen , Curr. Opin. Struct. Biol. 2014, 27, 24. 24709395 10.1016/j.sbi.2014.03.004 PMC5656570

[17] D. P. Karothu , Z. Alhaddad , C. R. Göb , C. J. Schürmann , R. Bücker , P. Naumov , Angew. Chem., Int. Ed. 2023, 62, e202303761. 10.1002/anie.202303761 37071841

[18] R. Vardanyan , V. Hruby , Synthes. Essential Drugs 2006, 219.

[19] M. Church , D. Church , Indian J. Dermatol. 2013, 58, 219. 23723474 10.4103/0019-5154.110832 PMC3667286

[20] H. L. Haas , O. A. Sergeeva , O. Selbach , Physiol. Rev. 2008, 88, 1183. 18626069 10.1152/physrev.00043.2007

[21] A. Weerts , N. Pattyn , P. Van De Heyning , F. Wuyts , J. Psychopharmacol. 2014, 28, 655. 24346808 10.1177/0269881113516201

[22] J. H. Day , A. K. Ellis , E. Rafeiro , Drugs Today 2004, 40, 415. 10.1358/dot.2004.40.5.850489 15319796

[23] C. G. Jones , M. W. Martynowycz , J. Hattne , T. J. Fulton , B. M. Stoltz , J. A. Rodriguez , H. M. Nelson , T. Gonen , ACS Cent. Sci. 2018, 4, 1587. 30555912 10.1021/acscentsci.8b00760 PMC6276044

[24] J. Hattne , M. W. Martynowycz , P. A. Penczek , T. Gonen , IUCrJ 2019, 6, 921. 10.1107/S2052252519010583 PMC6760445 31576224

[25] W. Kabsch , Acta Crystallogr. D. 2010, 66, 125. 20124692 10.1107/S0907444909047337 PMC2815665

[26] W. Kabsch , Acta Crystallogr. D. 2010, 66, 133. 20124693 10.1107/S0907444909047374 PMC2815666

[27] G. M. Sheldrick , Acta Crystallogr. A. 2015, 71, 3.

[28] Crystallographic Information File (CIF) of Meclizine dihydrochloride is deposited in Cambridge Crystallographic Data Center (CCDC) and the number is 2293443.

[29] G. M. Sheldrick , Acta Crystallogr. C. 2015, 71, 3.

[30] J.‐A. Van Den Berg , K. R. Seddon , Cryst. Growth Des. 2003, 3, 643.

[31] C. R. Martinez , B. L. Iverson , Chem. Sci. 2012, 3, 2191.

[32] R. Xia , N. Wang , Z. Xu , Y. Lu , J. Song , A. Zhang , C. Guo , Y. He , Nat. Commun. 2021, 12, 2086. 33828102 10.1038/s41467-021-22427-2 PMC8027608

[33] J. Eberhardt , D. Santos‐Martins , A. F. Tillack , S. Forli , J. Chem. Inf. Model. 2021, 61, 3891. 34278794 10.1021/acs.jcim.1c00203 PMC10683950

[34] O. Trott , A. J. Olson , J. Comput. Chem. 2010, 31, 455. 19499576 10.1002/jcc.21334 PMC3041641

[35] C. Chen , Curr. Med. Chem. 2008, 15, 2173. 18781943 10.2174/092986708785747625

[36] M. Gillard , C. Van Der Perren , N. Moguilevsky , R. Massingham , P. Chatelain , Mol. Pharmacol. 2002, 61, 391. 11809864 10.1124/mol.61.2.391

