# Supplementary material for: Unraveling the Structure of Meclizine Dihydrochloride with MicroED
Source: Adv Sci (Weinh). 2023 Dec 3;11(6):2306435. doi: 10.1002/advs.202306435 (PMC10853755; doi:10.1002/advs.202306435)
Supplement: Supplementary file 1 — Supporting Information [file ADVS-11-2306435-s001.pdf]

ADVANCED SCIENCE
Open Access

Supporting Information

for *Adv. Sci.*, DOI 10.1002/advs.202306435

Unraveling the Structure of Meclizine Dihydrochloride with MicroED

*Jieye Lin, Johan Unge and Tamir Gonen**

Supporting Information

Unraveling the Structure of Meclizine Dihydrochloride with MicroED

Jieye Lin[1] Johan Unge[1] and Tamir Gonen[1,2,3]*

[1]Department of Biological Chemistry, University of California, Los Angeles, 615 Charles E. Young Drive South, Los Angeles, California 90095, United States

[2] Department of Physiology, University of California, Los Angeles, 615 Charles E. Young Drive South, Los Angeles, California 90095, United States

[3] Howard Hughes Medical Institute, University of California, Los Angeles, Los Angeles, California 90095, United States

[*] Corresponding Author T.G. tgonen@g.ucla.edu

Methods

Materials.

Meclizine dihydrochloride, (*R/S*)-1-[(4-chlorophenyl)(phenyl)methyl]-4-(3-methylbenzyl)piperazine, was commercially purchased from InvivoChem and used as received without further recrystallization.

Grid preparation.

Sample preparation followed the procedure as described previously.[1] One carbon-coated copper grid (400-mesh, 3.05 mm O.D., Ted Pella Inc.) was pretreated with glow-discharge plasma at 15 mA on the negative mode using PELCO easiGlow (Ted Pella Inc.) for 60s. Around 1 mg of powdery compounds were carefully weighed by a Mettler Toledo (XPR225DR) analytical balance and mixed with a grid in a 10 mL scintillation vial. After gently shaking the vial, the grid was removed and clipped using c-ring and autogrid clip (Thermo Fisher) at room temperature.

MicroED data collection.

The clipped grid was loaded in an aligned Thermo Fisher Talos Arctica Cryo-TEM (200 kV, ~0.0251 Å) at 100 K, equipped with a CetaD CMOS camera (4096 × 4096 pixels) and EPUD (Thermo Fisher) software.[1,2] Screening of size- and thickness-suitable microcrystals was done in the imaging mode (LM 210× and SA 3400×). The MicroED data was collected in the diffraction mode with 659 mm diffraction length (the calibrated sample-detector distance), 70 µm C2 aperture, and a 50 µm selected area (SA) aperture in the parallel beam condition (45.2% C2

intensity) which resulted in a beam size at approximately 1.4 µm. Typical data collection used a constant rotation rate of ~2° per second over an angular wedge of 80° from -40° to +40°, with 0.5s exposure time per frame. Crystal selected for MicroED data collection were isolated and calibrated to eucentric heights to maintain the crystals inside the beam during the rotation.

MicroED data processing.

The MicroED data was saved in mrc format and converted to smv format using the mrc2smv software (https://cryoem.ucla.edu/microed).[2] The converted frames were indexed and integrated by XDS.[3,4] Two selected datasets with the highest resolution were scaled and merged using XSCALE,[4] and intensities were converted to SHELX hkl format using XDSCONV.[4] The merged dataset showed 80.7% overall completeness, which can be *ab initio* solved by SHELXT[5] at a resolution of 0.96 Å (Table S1). The structure was refined by SHELXL[6] in Shelxle[7] as a graphical interference to yield the final MicroED structure (Figure 1 and Table S2).

Molecular Docking.

The ligand structures of **1R/1S** were extracted from the refined MicroED structure and saved as mol2 files. The structure of levocetirizine was downloaded from CSD database (CSD entries: KIMDOD) and transformed to mol2 files. The Cl^- anions, water molecules, and polar hydrogen atoms from amine in the piperazine ring (pKa ≈ 2.12 and 6.55)[8] or ethoxyacetate group (pKa ≈ 2.9)[9] were removed. Then the ligand structures were imported into AutoDock Tools 1.5.7,[10] where all active torsion bonds were made rotatable.

The Cryo-EM structure of histamine H1 receptor (PDB entry: 7DFL)[11] was retrieved from the Protein Data Bank (https://www.rcsb.org/) after removing the ligand and other protein molecules using Pymol 2.5.5.[12] Subsequently, hydrogen atoms and charges were computed and added using AutoDock Tools 1.5.7.[10]

A grid box measuring 18.75 Å × 18.75 Å × 18.75 Å with 0.375 Å spacing was positioned along the *x*-, *y*-, and *z*-axes. The grid center was determined based on the experimental ligand (histamine) position at coordinates (131.312, 132.360, 158.503), see Figure S3.

The AutoDock Vina 1.1.2 was used for molecular docking,[13,14] where all active torsion bonds in ligands were set to be flexible, and the receptor was set as a rigid model. The docked complex with the minimum binding energy was exported and analyzed by the Protein-Ligand Interaction Profiler (PLIP) web tool and Pymol 2.5.5 (Figure 3 and Tables S5-S8).[12,15]

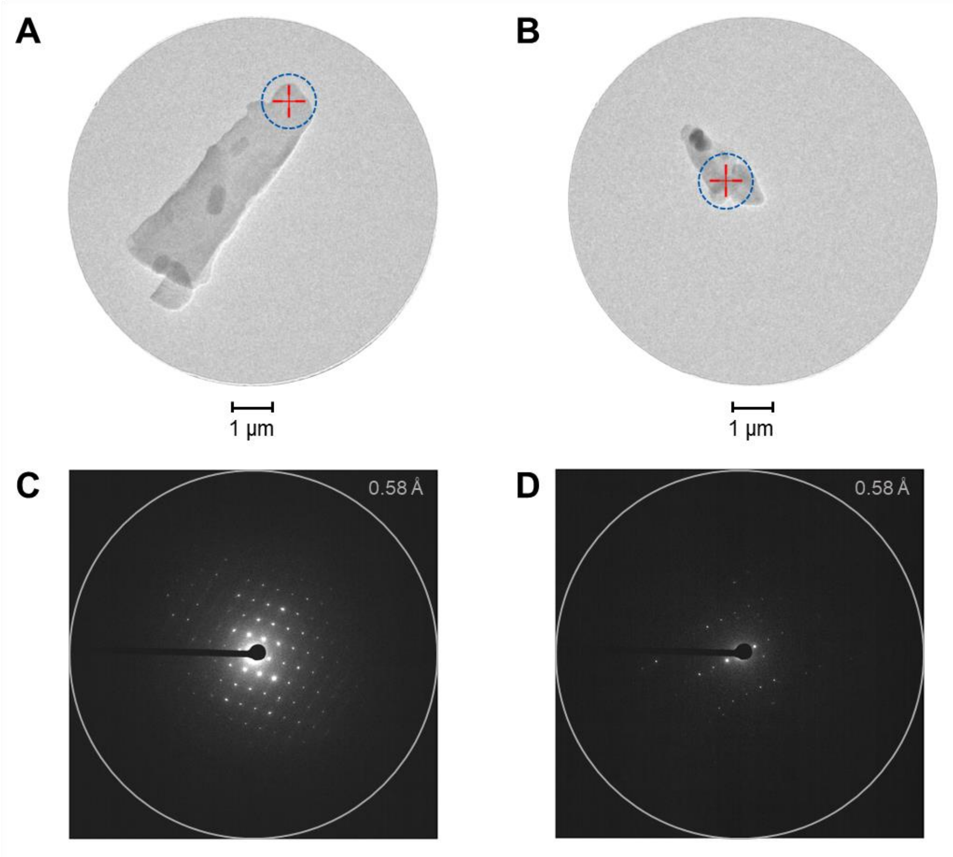

Figure S1 Crystal appearance and diffraction pattern under the TEM. (A-B) Images of items 1 and 2 under the imaging mode (SA 3400×), respectively. The diffraction beam size was highlighted in dashed blue circles; (C-D) Diffraction pattern of items 1 and 2 under diffraction mode (659 mm), respectively. The integration edge was colored in grey rings.

A

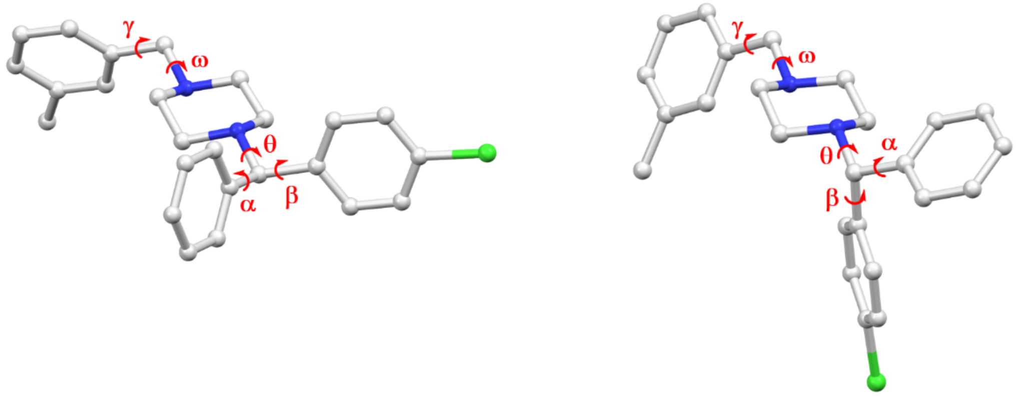

Crystal Structure:		Molecular Docking:	
α (N1-C13-C10-C9)	-46.44°	α (N1-C13-C10-C9)	-13.55°
β (N1-C13-C4-C5)	+47.58°	β (N1-C13-C4-C5)	+2.42°
θ (C10-C13-N1-C17)	-61.68°	θ (C10-C13-N1-C17)	-170.24°
ω (C16-N2-C18-C19)	-64.46°	ω (C16-N2-C18-C19)	-83.66°
γ (N2-C18-C19-C20)	-79.05°	γ (N2-C18-C19-C20)	-6.60°

B

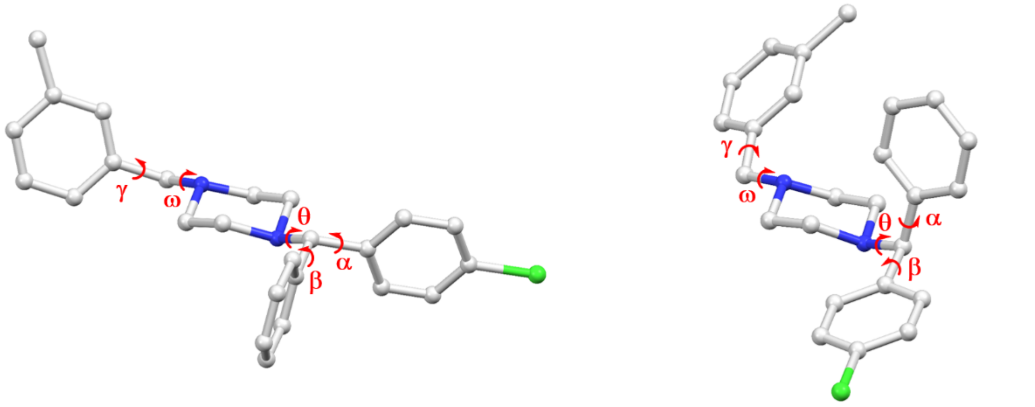

Crystal Structure:		Molecular Docking:	
α (N1'-C13'-C10'-C9')	+46.44°	α (N1'-C13'-C10'-C9')	+15.36°
β (N1'-C13'-C4'-C5')	-47.58°	β (N1'-C13'-C4'-C5')	-13.75°
θ (C10'-C13'-N1'-C17')	+61.68°	θ (C10'-C13'-N1'-C17')	-65.02°
ω (C16'-N2'-C18'-C19')	+64.46°	ω (C16'-N2'-C18'-C19')	+169.99°
γ (N2'-C18'-C19'-C20')	+79.05°	γ (N2'-C18'-C19'-C20')	-4.76°

Figure S2 The major conformation changes between the crystal structures of **1R/1S** and their molecular docking structures. See notations in Figure 1B.

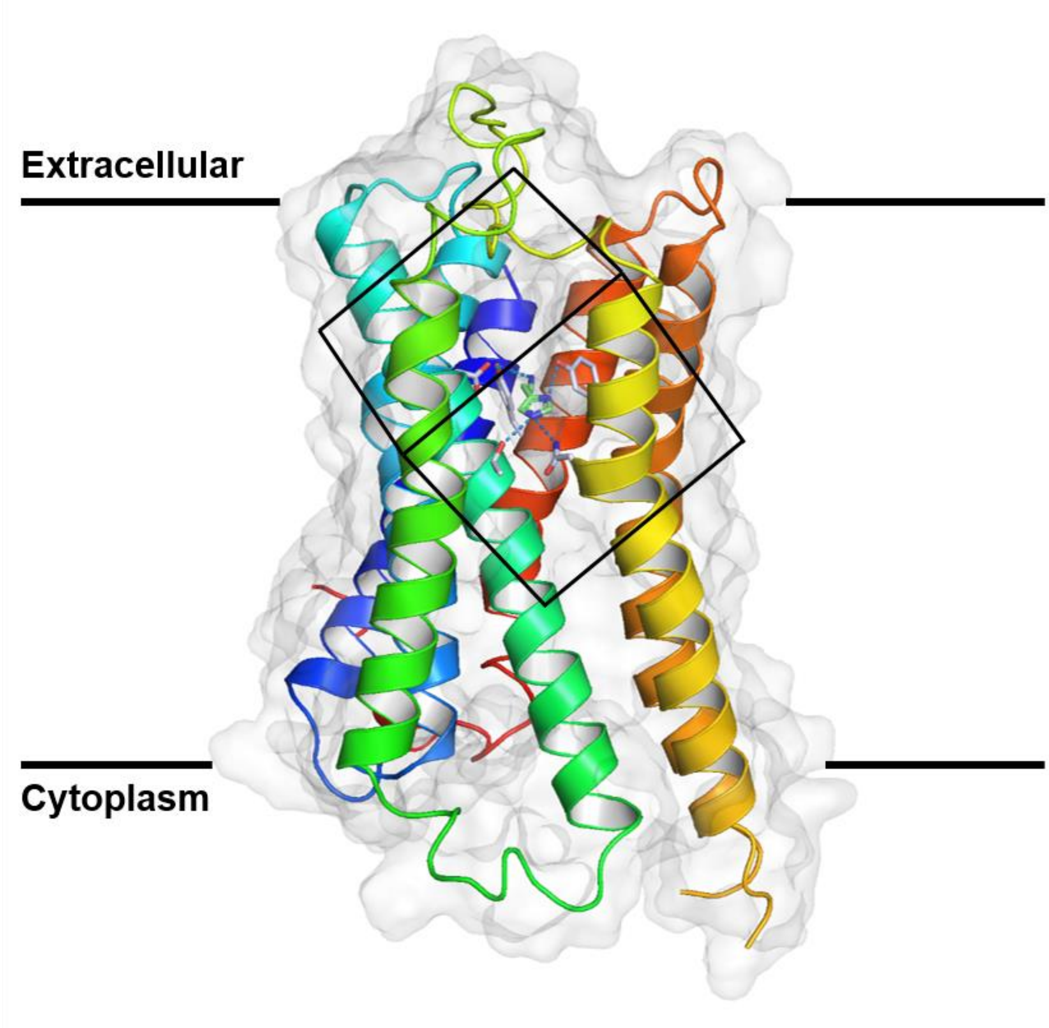

Figure S3 Overall view of the complex between histamine H1 receptor and histamine determined by Cryo-EM (PDB entry: 7DFL). The 18.75 Å × 18.75 Å × 18.75 Å grid box used for molecular docking was centered by the experimental ligand (histamine) position at coordinates (131.312, 132.360, 158.503), marked by black lines.

Table S1 MicroED data statistics of two selected items of meclizine dihydrochloride.

	Item 1	Item 2
Space group	$P2_1$	$P2_1$
Unit cell lengths (Å)		
a	14.39	14.00
b	7.19	7.17
c	24.52	24.41
Unit cell angles (°)		
α	90.000	90.000
β	100.958	103.276
γ	90.000	90.000
No. of observed reflections	4870	4845
No. of unique reflections	1869	1812
R_{obs} (%)	12.7	22.6
R_{meas} (%)	16.1	28.6
I/Sigma	5.14	3.51
$CC_{1/2}$	99.3	96.9
Resolution (Å)	0.95	0.95
Completeness (%)	55.8	56.3

Table S2 MicroED data statistics of meclizine dihydrochloride (merged).

Stoichiometric formula	$C_{25}H_{29}Cl_3N_2$
Mr	463.85
Temperature (K)	100
Crystal system	Monoclinic
Space group	$P2_1/c$
Unit cell lengths (Å)	
a	14.39
b	7.19
c	24.52
Unit cell angles (°)	
α	90.000
β	101.958
γ	90.000
Cell volume (Å^3)	2493.49
No. of observed reflections	9487
No. of unique reflections	2623
R_{obs} (%)	19.0
R_{meas} (%)	22.3
I/Sigma	3.81
$CC_{1/2}$	98.9
Resolution (Å)	0.96
Completeness (%)	**80.7**
R_1 (%)	**17.89**
wR_2 (%)	42.43
GooF	1.737

Table S3 Hydrogen-bond geometry in meclizine dihydrochloride **1R/1S** (Å, °).

**R form**	**D–H**	**H···A**	**D···A**	**D–H···A**	**Type**
N1–H···Cl1	1.008	2.041	3.013	161.27	N–H···Cl^-
N2–H···Cl2	1.117	1.877	2.994	178.15	
C3–H···Cl1^{i}	0.930	2.711	3.473	139.75	C–H···Cl^-
C5–H···Cl1	0.930	2.804	3.679	157.28	
C9–H···Cl1	0.930	2.726	3.593	155.60	
C13–H···Cl1^{i}	0.981	2.677	3.598	156.45	
C15–Ha···Cl1^{ii}	0.969	2.754	3.709	168.59	
C15–Hb···Cl1	0.971	2.933	3.647	131.29	
C14–H···Cl2^{iv}	0.969	2.765	3.569	140.75	
C18–H···Cl2^{iii}	0.972	2.656	3.605	165.30	
C24–H···Cl2^{iii}	0.931	2.900	3.719	147.42	
C25–H···Cl3^{ii}	0.961	2.921	3.570	125.90	C–H···Cl
**S form**	**D–H**	**H···A**	**D···A**	**D–H···A**	**Type**
N1′–H···Cl1′	1.008	2.041	3.013	161.27	N–H···Cl^-
N2′–H···Cl2′	1.117	1.877	2.994	178.15	
C3′–H···Cl1′^{iii}	0.930	2.711	3.473	139.75	C–H···Cl^-
C5′–H···Cl1′	0.930	2.804	3.679	157.28	
C9′–H···Cl1′	0.930	2.726	3.593	155.60	
C13′–H···Cl1′^{iii}	0.981	2.677	3.598	156.45	
C15′–Ha···Cl1′^{v}	0.969	2.754	3.709	168.59	
C15′–Hb···Cl1′	0.971	2.933	3.647	131.29	
C14′–H···Cl2′^{vi}	0.969	2.765	3.569	140.75	
C18′–H···Cl2′^{i}	0.972	2.656	3.605	165.30	
C24′–H···Cl2′^{i}	0.931	2.900	3.719	147.42	
C25′–H···Cl3′^{ii}	0.961	2.921	3.570	125.90	C–H···Cl

Symmetry codes: (i) x,-1+y,z; (ii) 1-x,-1/2+y,1.5-z; (iii) x,1+y,z; (iv) 1-x,1/2+y,1.5-z; (v) 1-x,1/2+y,1/2-z; (vi) 1-x,-1/2+y,1/2-z.

Notes: See details in Figure 2.

Table S4 Pi-stacking interactions in meclizine dihydrochloride **1R/1S** (Å, °).

pi-stacking interactions in 1R				
**Centroid 1**[a]	**Centroid 2**[a]	**Distance**[b]	**Relative Orientation**[c]	**Type**
1	4	**4.58**	19.22	Parallel-displaced
	5	5.65	12.57	Parallel-displaced
	6	5.65	12.57	Parallel-displaced
	7	6.6	19.22	Parallel-displaced
2	8	**4.85**	74.69	T-shaped
	9	5.59	74.69	T-shaped
	10	6.09	74.69	T-shaped
3	11	**4.58**	19.22	Parallel-displaced
	12	**4.85**	74.69	T-shaped
	13	5.59	74.69	T-shaped
	14	6.09	74.69	T-shaped
	15	6.50	0	Parallel-displaced
	16	6.59	0	Parallel-displaced
	17	6.60	19.22	Parallel-displaced
pi-stacking interactions in 1S				
**Centroid 1**[a]	**Centroid 2**[a]	**Distance**[b]	**Relative Orientation**[c]	**Type**
1′	4′	**4.58**	19.22	Parallel-displaced
	5′	5.65	12.57	Parallel-displaced
	6′	5.65	12.57	Parallel-displaced
	7′	6.6	19.22	Parallel-displaced
2′	8′	**4.85**	74.69	T-shaped
	9′	5.59	74.69	T-shaped
	10′	6.09	74.69	T-shaped
3′	11′	**4.58**	19.22	Parallel-displaced
	12′	**4.85**	74.69	T-shaped
	13′	5.59	74.69	T-shaped
	14′	6.09	74.69	T-shaped
	15′	6.50	0	Parallel-displaced
	16′	6.59	0	Parallel-displaced
	17′	6.60	19.22	Parallel-displaced

Notes: [a]Centroids were determined as the center of aromatic phenyl rings in **1R**/**1S**. [b]Distances were measured between centroids.[c]Relative orientations were measured by the angles between planes of two aromatic rings. See details in Figure 2.

Table S5 Protein-ligand interactions of histamine H1 receptor and histamine determined by Cryo-EM (Å, °).

**Hydrogen Bonds**					
Residue	AA	Ligand Group[a]	H–A	D–A	D-H···A
107R	ASP	$EtNH_2$	2.41	3.38	158.95
458R	TYR	$EtNH_2$	3.16	3.94	139.55
112R	THR	Im	2.35	3.07	127.05
198R	ASN	Im	2.47	3.09	118.28
431R	TYR	Im	2.18	3.02	147.81
**Hydrophobic Interactions**					
Residue	AA	Ligand Group[a]	Distance		
108R	TYR	$EtNH_2$	3.75		
431R	TYR	$EtNH_2$	3.82		

Notes: [a]Ligand group was represented by abbreviation: imidazole (Im), ethylamine ($EtNH_2$).

Table S6 Protein-ligand interactions of histamine H1 receptor and **1R** complex predicted by molecular docking (Å, °).

**Salt Bridges**					
Residue	AA	Distance	Protein positive?	Ligand Group[a]	
107R	ASP	3.76	No	Pip	
107R	ASP	3.69	No	Pip	
**Hydrogen Bonds**					
Residue	AA	Ligand Group[a]	H–A	D–A	D–H···A
458R	TYR	Pip	3.30	4.00	132.70
**π-Stacking**					
Residue	AA	Ligand Group[a]	Distance[b]	Angle[c]	Stacking Type
103R	TRP	Ph	4.62	66.31	T-shaped
450R	HIS	Ph	4.03	71.31	T-shaped
450R	HIS	ClPh	3.79	65.97	T-shaped
**Hydrophobic Interactions**					
Residue	AA	Ligand Group[a]	Distance		
454R	ILE	Ph	3.90		
87R	TYR	Ph	3.19		
454R	ILE	Ph	3.44		
108R	TYR	ClPh	3.94		
182R	THR	ClPh	2.56		
108R	TYR	MeBn	3.69		
115R	ILE	MeBn	3.62		
158R	TRP	MeBn	3.46		
428R	TRP	MeBn	3.33		
428R	TRP	MeBn	3.97		
431R	TYR	MeBn	3.47		
432R	PHE	MeBn	3.18		

Notes: [a]Ligand group was represented by abbreviation: piperazine (Pip), phenyl (Ph), chlorophenyl (ClPh), methylbenzyl (MeBn). [b]Distances were measured between centroids determined as the center of aromatic rings. [c]Angles were measured by the angles between planes of two aromatic rings.

Table S7 Protein-ligand interactions of histamine H1 receptor and **1S** complex predicted by molecular docking (Å, °).

**Salt Bridges**					
Residue	AA	Distance	Protein positive?	Ligand Group[a]	
107R	ASP	3.68	No	Pip	
107R	ASP	3.79	No	Pip	
**Hydrogen Bonds**					
Residue	AA	Ligand Group[a]	H–A	D–A	D–H···A
458R	TYR	Pip	3.33	4.08	137.47
**π-Stacking**					
Residue	AA	Ligand Group[a]	Distance[b]	Angle[c]	Stacking Type
450R	HIS	Ph	3.80	70.65	T-shaped
103R	TRP	ClPh	4.74	65.54	T-shaped
450R	HIS	ClPh	3.90	73.94	T-shaped
432R	PHE	MeBn	4.83	70.17	T-shaped
**Hydrophobic Interactions**					
Residue	AA	Ligand Group[a]	Distance		
108R	TYR	Ph	3.84		
182R	THR	Ph	2.56		
431R	TYR	Ph	3.80		
87R	TYR	ClPh	3.45		
454R	ILE	ClPh	3.18		
454R	ILE	ClPh	3.69		
108R	TYR	MeBn	3.71		
115R	ILE	MeBn	3.69		
158R	TRP	MeBn	3.54		
428R	TRP	MeBn	3.84		
428R	TRP	MeBn	3.39		
431R	TYR	MeBn	3.25		

Notes: [a]Ligand group was represented by abbreviation: piperazine (Pip), phenyl (Ph), chlorophenyl (ClPh), methylbenzyl (MeBn). [b]Distances were measured between centroids determined as the center of aromatic rings. [c]Angles were measured by the angles between planes of two aromatic rings.

Table S8 Protein-ligand interactions of histamine H1 receptor and levocetirizine complex predicted by molecular docking (Å, °).

Salt Bridges

Residue	AA	Distance	Protein positive?	Ligand Group[a]
107R	ASP	3.76	No	Pip
107R	ASP	3.73	No	Pip

Hydrogen Bonds

Residue	AA	Ligand Group[a]	H–A	D–A	D–H···A
198R	ASN	EtOAc	2.25	2.98	126.87
431R	TYR	EtOAc	2.28	3.10	144.14
431R	TYR	EtOAc	1.92	2.82	158.91
458R	TYR	Pip	3.28	4.03	136.96

π-Stacking

Residue	AA	Ligand Group[a]	Distance[b]	Angle[c]	Stacking Type
103R	TRP	Ph	4.54	71.57	T-shaped
450R	HIS	Ph	4.08	71.58	T-shaped
450R	HIS	ClPh	3.87	72.53	T-shaped

Halogen Bonds

Residue	AA	Ligand Group[a]	**X**···Y	A–**X**···Y	**X**···Y–B
191R	LYS	ClPh	3.02	140.96	138.51

Hydrophobic Interactions

Residue	AA	Ligand Group[a]	Distance
87R	TYR	Ph	3.22
454R	ILE	Ph	3.78
454R	ILE	Ph	3.95
108R	TYR	ClPh	3.87
182R	THR	ClPh	2.72
431R	TYR	ClPh	3.61

Notes: [a]Ligand group was represented by abbreviation: piperazine (Pip), phenyl (Ph), chlorophenyl (ClPh), ethoxyacetic acid (EtOAc). [b]Distances were measured between aromatic ring centroids or centroid-cation. [c]Angles were measured by the angles between planes of two aromatic rings.

Reference

1 C. G. Jones, M. W. Martynowycz, J. Hattne, T. J. Fulton, B. M. Stoltz, J. A. Rodriguez, H. M. Nelson, T. Gonen, *ACS Cent. Sci.* **2018**, *4*, 1587-1592.

2 J. Hattne, M. W. Martynowycz, P. A. Penczek, T. Gonen, *IUCrJ* **2019**, *6*, 921-926.

3 W. Kabsch, *Acta Crystallogr. Sect. D.* **2010**, *66*, 125-132.

4 W. Kabsch, *Acta Crystallogr. Sect. D.* **2010**, *66*, 133-144.

5 G. M. Sheldrick, *Acta Crystallogr. Sect. A.* **2015**, *71*, 3-8.

6 G. M. Sheldrick, *Acta Crystallogr. Sect. C.* **2015**, *71*, 3-8.

7 C. B. Hübschle, G. M. Sheldrick, B. Dittrich, *J. Appl. Crystallogr.* **2011**, *44*, 1281-1284.

8 M. Bitencourt, O. M. M. S. Viana, A. L. M. Viana, J. T. J. Freitas, C. C. de Melo, A. C. Doriguetto, *Int. J. Pharm.* **2020**, *589*, 119840.

9 H.-R. Park, S. H. Seok, E.-S. Park, *J. Pharm. Investig.* **2023**, *53*, 377-388.

10 S. Forli, R. Huey, M. E. Pique, M. F. Sanner, D. S. Goodsell, A. J. Olson, *Nat. Protoc.* **2016**, *11*, 905-919.

11 R. Xia, N. Wang, Z. Xu, Y. Lu, J. Song, A. Zhang, C. Guo, Y. He, *Nat. Commun.* **2021**, *12*, 2086.

12 PyMol, *Schrödinger. LLC* **2017**.

13 J. Eberhardt, D. Santos-Martins, A. F. Tillack, S. Forli, *J. Chem. Inf. Model.* **2021**, *61*, 3891-3898.

14 O. Trott, A. J. Olson, *J. Comput. Chem.* **2010**, *31*, 455-461.

15 M. F. Adasme, K. L. Linnemann, S. N. Bolz, F. Kaiser, S. Salentin, V. J. Haupt, M. Schroeder, *Nucleic Acids Res.* **2021**, *49*, W530-W534.

16 A. Daina, O. Michielin, V. Zoete, *J. Chem. Inf. Model.* **2014**, *54*, 3284-3301.

17 A. Daina, O. Michielin, V. Zoete, *Sci. Rep.* **2017**, *7*, 42717.
